# Plasma Phospholipid Biomarkers Related to the Risk of Cognitive Decline in the Elderly: Results from a Cohort Study

**DOI:** 10.3390/nu18020185

**Published:** 2026-01-06

**Authors:** Ting-Ting Liu, Jia-Wei Xie, Xin Long, Xin-Can Yu, Shan-Shan Jia, Qing-Qing Man, Jing Li, Pu-Jun Quan, Ke-Chang Shan, Jian Zhang, Shuang Song, Dan Liu

**Affiliations:** 1 National Institute for Nutrition and Health, Chinese Center for Disease Control and Prevention, Beijing 100050, China; 2 Department of Epidemiology, School of Public Health, Southern Medical University, Guangzhou 510515, China; 3 Department of Public Health and Preventive Medicine, School of Medicine, Jinan University, Guangzhou 510632, China; 4 Key Laboratory of Public Nutrition and Health, National Health Commission of the People’s Republic of China, Beijing 100050, China

**Keywords:** phospholipid, biomarker, cognitive decline risk, cohort study

## Abstract

Phospholipids provide both structural and functional varieties for neuro cells, and their dysregulation in brain has been related to pathogenesis of cognitive impairment. The reflection of these phospholipid alterations in the blood might serve as biomarkers for the early recognition of cognitive decline risk preceding clinical symptoms and provide potential targets for intervention. In this cohort study, detailed phospholipid molecular profiles including 229 species were quantified. A total of 209 participants aged 60–80 years (including 138 women and 73 men) were followed for one year, during which 32 participants developed significant cognitive decline, defined as a decrease of three or more points in the Montreal Cognitive Assessment score. A biomarker panel of eight phospholipid molecular species related to cognitive decline was identified by Least Absolute Shrinkage and Selection Operator (LASSO) logistic regression between cases and non-cases. Among these, four biomarkers, including PE(O-40:5), LPC(18:3), PI(38:2) and PA(39:4), were further proved to be significantly associated with the risk of cognitive decline through a logistic regression model, indicating that the degradation of phospholipids and the accumulation of ether phospholipid and PI might participate in the process of cognitive decline in early stage. By adding the eight phospholipid biomarkers to a reference model that included demographics, lifestyle, hypertension, fasting blood glucose and blood lipid parameters, the AUC value of the predictive model improved from 0.743 to 0.866, which provided a possible auxiliary screening tool for the early detection of cognitive impairment in the elderly.

## 1. Introduction

Cognitive decline in the elderly is becoming a serious problem among the aging societies around the world and extends far beyond simple memory lapses, posing a profound and multi-faceted threat to well-being, autonomy, and safety [1,2]. Despite its prevalence, the precise mechanistic pathways underlying cognitive decline in the elderly remain largely enigmatic, presenting a formidable challenge to the development of effective treatments [3]. The brain is an organ with exceptionally high metabolic demands. Alterations in metabolic balance can compromise brain function, driving structural damage and cognitive deficits. The identification of metabolic changes associated with cognitive decline in early stages is critical for both understanding the underlying pathogenesis and early diagnosis/intervention of cognitive impairment.

As the most abundant building blocks of the brain’s intricate architecture, phospholipids not only provide structural and functional variety for the membrane of neural cells [4,5], but also participate in the conduction of neurotransmitter and electrical signals [6]. Based on the molecular scaffold to which the fatty acid tails and phosphate group are attached, phospholipids could be classified into glycerophospholipids and sphingophospholipids. The former could be further divided into phosphatidylcholine (PC), phosphatidylethanolamines (PE), phosphatidylserine (PS), phosphatidylinositol (PI), phosphatidylglycerol (PG) and phosphatidic acid (PA), while the latter mainly refers to sphingomyelin (SM).

Over the past decade, a growing body of evidence underscores the critical role of brain phospholipid dysregulation in the pathogenesis of cognitive impairment, spanning from age-related cognitive decline to Alzheimer’s disease (AD) and other dementias. For instance, the reduction in PI and PE levels in dementia brain is consistent in most studies, while decreased or unchanged PC and SM levels were observed in different studies [7,8]. Meanwhile, decreased content of plasmenyl phosphatidylethanolamine (pPE, plasmalogen sub-class of PE) and plasmenyl phosphatidylcholine (pPC, plasmalogen sub-class of PC) were reported in both white and gray matter of dementia patients [9,10]. The reflection of these phospholipid alterations in peripheral blood preceding clinical symptoms might serve as minimally invasive biomarkers for the early recognition of cognitive decline and provide potential targets for intervention.

The central challenge in the validation of phospholipids as reliable biomarkers lies in their precise molecular characterization. But the structural complexity of phospholipids precludes simple quantification. Thus, most studies only focused on a few classes of plasma phospholipid, like total pPE [7] or plasma PC and SM [8,9]. In our previous study, an LC-MS/MS method was developed for the characterization of detailed phospholipid profiles in plasma and used to identify the association of plasma phospholipids with cognitive impairment a cross-sectional study. Although 23 phospholipid molecular species were found to be associated with Montreal Cognitive Assessment (MoCA) score independent of fasting glucose, lipidemia, lipoproteins, inflammatory variables and homocysteine, these phospholipid molecules could not predict the risk of cognitive impairment due to the cross-sectional design.

Thus, in this study, detailed phospholipid molecular profiles were identified and quantified in a cohort study, which followed 209 participants aged 60–80 years for one year, and found 32 cases with significant cognitive decline. The relationship between plasma phospholipids and the risk of cognitive decline was discussed with related lifestyle, hypertension, blood glucose and blood lipid factors considered and controlled stepwise to find a reliable biomarker panel that could help to predict cognitive decline.

## 2. Materials and Methods

### 2.1. Study Participants and Design

**Figure 1:**
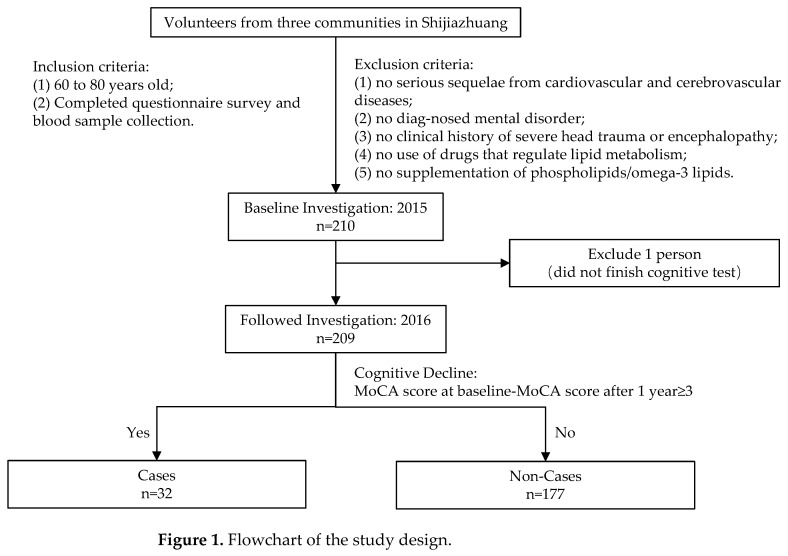
Flowchart of the study design.

The participants of this study were volunteers from three communities (Yuxing, Qingyuan and Jianxin community) located in Shijiazhuang, China. The inclusion criteria are as follows: (1) 60 to 80 years old; (2) completed the questionnaire survey and blood sample collection. The exclusion criteria are the following: (1) no serious sequelae from cardiovascular and cerebrovascular diseases; (2) no diagnosed mental disorder; (3) no clinical history of severe head trauma or encephalopathy; (4) no use of drugs that regulate lipid metabolism (including statins, aspirin, GLP-1 receptor agonists, thiazolidinediones, metformin and traditional Chinese medicines for regulating blood lipids); (5) no supplementation of phospholipids/omega-3 lipids. A total of 210 residents were included in baseline investigation conducted in 2015. After 1 year, all these participants were followed up and eventually 209 finished the investigation (Figure 1). For all participants, demographic information, cognitive assessment and fasting blood samples were collected at both baseline and follow-up. Blood lipid parameters and plasma phospholipid profile were analyzed among baseline blood samples. According to the principles of the Declaration of Helsinki, the study protocol was approved by the ethics committee of the National Institute of Nutrition and Health, Chinese Center for Disease Control and Prevention (No. 2013-013, 4 March 2013). Written informed consent was gathered from all participants prior to investigation.

### 2.2. Demographic and Lifestyle Variables

Basic demographic and lifestyle information was obtained according to the questionnaire at baseline, including sex, age, educational attainment, smoking status, physical activity and sleep duration.

### 2.3. Condition of Hypertension, Diabetes Mellitus and Dyslipidemia

The hypertension condition was obtained according to the questionnaire. The status of diabetes mellitus and dyslipidemia were identified through fasting blood glucose (Glu) and blood lipid biochemical parameters, including total cholesterol (TC), triglycerides (TG), low-density lipoprotein cholesterol (LDL-C), and high-density lipoprotein cholesterol (HDL-C) obtained in the baseline investigation. Diabetes mellitus was diagnosed at Glu ≥ 7.0 mmol/L [10]. And dyslipidemia was diagnosed at TC ≥ 6.2 mmol/L, TG ≥ 2.3 mmol/L, LDL-C ≥ 4.1 mmol/L or HDL-C < 1.0 mmol/L, according to the stratification criteria for dyslipidemia in the primary prevention of ASCVD in China.

### 2.4. Cognitive Decline Assessment

Cognitive function was evaluated through Montreal Cognitive Assessment (MoCA) at both baseline and 1-year follow-up. The MoCA questionnaire has a score range of 0–30, calculated from 52 items that cover 8 cognitive domains (visuospatial/executive ability, naming, memory, attention, language, abstraction, delayed recall and orientation). Lower MoCA scores signify more impairment. Individuals with MoCA score decreased by 3 or more points within one year were classified as the cognitive decline cases, while the other individuals were defined as non-cases (Figure S1).

### 2.5. Plasma Collection and Storage

In the baseline investigation, fasting blood samples were collected from all the participants using EDTA-K2 blood tubes (BD, Franklin Lakes, NJ, USA). All the samples were centrifuged at 800× *g* for 30 min at 4 °C. The upper layer of plasma was transferred into sterile tubes and stored at −80 °C before analysis.

### 2.6. Analysis of Biochemical Parameters

Fasting blood glucose (Glu) and lipid biochemical parameters, including total cholesterol (TC), triglycerides (TG), low-density lipoprotein cholesterol (LDL-C) and high-density lipoprotein cholesterol (HDL-C) at baseline were analyzed with an enzymatic method using a Hitachi 7600 automatic biochemical analyzer (Hitachi, Tokyo, Japan).

### 2.7. Analysis of Plasma Phospholipid Profile

Total lipid was extracted from human plasma according to a modified Folch method as reported in our previous study [11]. The phospholipid molecular species were quantified according to a hydrophilic liquid chromatography-electrospray ionization-triquadrupole-mass spectrometry (HILIC-ESI-MS/MS) method as described and validated in our previous research [12]. A targeted MRM mode was used to collect the MS/MS data for all the phospholipid molecular species. All the plasma samples were analyzed in random order with QC samples (mixed plasma from over 1000 people prepared in our lab) and blank samples (methanol solvent) added after every 10 samples to evaluate stability and ensure no carryover of lipids in the column. Internal standard method was used to quantify each phospholipid molecular species. The standards and internal standards (Avanti Polar Lipids, Alabaster, AL, USA) used for quantification were listed in Table S1. A total of 229 phospholipids were detected in >50% plasma samples, including 62 PCs, 55 PEs, 30 SMs, 22 PSs, 19 PIs, 5 PGs, 3 Pas, 22 LPCs and 11 LPEs (as listed in Table S2).

### 2.8. Statistical Analysis

The undetected phospholipid concentrations were assigned a value as half of the detection limit. Phospholipid concentration values that exceeded the mean by ±3 standard deviations were retested, and the average of the two tests was used for subsequent analysis. Descriptive data were described as means and standard deviations (SDs) for continuous variables, and as frequencies with percentages for categorical variables. Baseline characteristics were compared between groups using chi-square tests, Student’s t-test or Wilcoxon test where appropriate using R (version 4.5.2). Before conducting further statistical analysis, logarithmic transformation and UV scaling were applied to standardize the variance of the phospholipidomics data and reduce the impact of outliers. Principal component analysis of phospholipid molecular species in participants’ plasma and QC samples was conducted using SIMCA software (version 14.1, MKS Umetrics, Umeå, Sweden). The Least Absolute Shrinkage and Selection Operator (LASSO) logistic regression method in the ‘glmnet’ package of R (version 4.5.2) was used to identify potential phospholipid molecules associated with cognitive decline. The model complexity was reduced through L1 regularization, the regularization parameter (λ) was optimized using k-fold cross-validation (10-fold in this study), and the value with the smallest error was selected to ensure robust variable selection. A collinearity diagnosis was further conducted to exclude phospholipid molecules with variance inflation factors (VIFs) > 10. Bootstrap resampling was employed for stability analysis to reduce the random bias of a single LASSO regression. A total of 800 pseudo-training sets were generated through 800 resamples with the same sample size as the original dataset. LASSO regression was performed on each pseudo-training set, using 10-fold cross-validation to determine the regularization parameter λ. Multivariate logistic analysis was used to analyze the associations of potential phospholipid biomarkers with cognitive decline. Phospholipid levels were divided into quartiles, and the first quartile group was applied as the reference. Restricted cubic spline models with 3 knots at the 10th, 50th, and 90th percentiles were used to assess the dose–response associations of plasma phospholipid molecules with cognitive decline. Linearity was assessed using the Wald test. The performance of regression models with or without phospholipid biomarkers in prediction of cognitive decline risk was tested. Net reclassification index (NRI) and integrated discrimination improvement (IDI) were calculated to evaluate the improvement in risk classification by adding potential phospholipid biomarkers to the reference model. The Hosmer−Lemeshow test was used to assess the goodness of fit of the model. Receiver operating characteristic (ROC) curve with area under the ROC curve (AUC) and calibration curve were used to evaluate the predictive accuracy and conformity of the model. Decision curve analysis (DCA) was used to reflect the net benefit of the model.

## 3. Results

### 3.1. General Characteristics of Participants

In the present study, a total of 209 participants were included, the median age was 65.8 years (range from 60 to 80), of which 138 (66.0%) were female, and 200 (95.7%) had a primary or above educational attainment (Table 1). Over 1 year, 31 (14.8%) participants showed a significant decline in cognitive function (MoCA score decreased by three or more points). No significant differences were observed between the cases and non-cases in terms of sex, age, educational attainment, smoking status, physical activity and sleep duration. And there was no statistically significant difference in the number of people with hypertension, diabetes mellitus and dyslipidemia between the case group and the non-case group at baseline. Among the blood lipid parameters, TC, TG and LDL-C showed no significant differences between the cases and non-cases, but cases were more likely to have lower HDL-C values (*p* = 0.051). At baseline, the MoCA scores of the cases (24.8 in average) were slightly higher than those of non-cases (23.3 in average). After one year, the MoCA scores of the cases decreased to an average of 20.7, while the MoCA scores of the non-cases increased to an average of 25.3 (probably due to the practice effect).

### 3.2. Characteristics of Plasma Phospholipids

A total of 229 phospholipid molecular species were identified and quantified, including 62 PCs, 55 PEs, 30 SMs, 22 PSs, 19 PIs, 5 PGs, 3 PAs, 22 LPCs and 11 LPEs. A PCA model was established by the phospholipidome data matrix of all the participants’ plasma samples and QC samples to evaluate the stability of quantitative analysis. In score plot of PCA model (Figure S2), all the QC samples were well-gathered, indicating that the quantification of phospholipid molecular species was stable. The concentration of different phospholipid classes was listed in Table 2. Within the phospholipid classes, PC had the highest concentrations, followed by SM, LPC, PE, PI, LPE and PS. PG and PA had concentrations lower than 1 mg/L. Compared with non-cases, cases had slightly higher concentration of SM (*p* = 0.082), while other phospholipid classes showed no significant differences between cases and non-cases.

### 3.3. Identification of Potential Phospholipid Biomarkers Related with Cognitive Decline by Machine Learning

The 229 phospholipid molecular species were introduced into LASSO model to identify phospholipid molecules that were probably related to cognitive decline. The 10-fold cross-validation method was applied to the iterative analysis. According to the LASSO path diagram, the number of predictor variables decreased along with the coefficients (Figure S3). A model with excellent performance but minimum number of variables was obtained when λ was 0.014 (Log_e_λ = −4.25). And 17 phospholipid molecules with coefficients not equal to 0 were included in the final model, including PE(P-44:5), PE(O-40:5), PE(36:3), PC(42:4), PC(35:1), PC(O-32:0), SM(d36:2), SM(d32:2), PS(34:3), PS(40:6), LPC(20:2), LPC(18:3), PA(39:9), PG(36:3), PG(34:2), PI(38:2), and PI(36:1). Among them, PE(P-44:5) is a kind of plasmalogen having vinyl ether linkage at the *sn*-1 position of glycerol backbone, while PE(O-40:5) and PC(O-32:0) are ether phospholipids having ether linkage at the *sn*-1 position of glycerol backbone. The others are diacyl-phospholipids with ester bonds at both *sn*-1 and *sn*-2 positions of their glycerol backbone. Furthermore, collinearity was diagnosed for these phospholipid molecules; PC(35:1) was excluded due to a variance inflation factor (VIF) > 10 (Table S3). The reliability and reproducibility of the remaining 16 phospholipid molecules were further evaluated by bootstrap analysis with LASSO regressions in 800 pseudo-training sets. The phospholipid variables selected for inclusion in each model were recorded. And the selection frequency of each phospholipid variable across the 800 samples were calculated (Table S4). A total of eight phospholipid variables with a selection frequency exceeding 0.5, including PE(O-40:5), PC(42:4), PS(34:3), LPC(18:3), PA(39:4), PG(36:3), PG(34:2) and PI(38:2), were selected as potential biomarkers related with cognitive decline and included in subsequent analyses.

### 3.4. Associations Between Phospholipid Biomarkers with Cognitive Decline

The associations between the potential phospholipid biomarkers and cognitive decline were further analyzed through logistic regression. The demographic, lifestyle, hypertension, fasting blood glucose and fasting blood lipid biochemical variables were diagnosed for collinearity. Only TC was excluded to avoid overadjustment, since it could be estimated from LDL-C, HDL-C and TG using the Friedewald formula. All the variables showed VIF < 10 (Table S5) and were stepwise included in the logistic regression model as covariates. As shown in Table 3, with the adjustment of sex and age, higher levels of PG(36:3) were associated with a lower risk of cognitive decline (*p* < 0.05), while higher levels of PE(O-40:5), LPC(18:3), PI(38:2) and PA(39:4) were associated with a higher risk of cognitive decline (*p* < 0.05). Further adjustment of educational attainment, smoking status, physical activity and sleep duration did not change the significances of the associations between phospholipid molecules and cognitive decline. With the adjustment of hypertension condition, fasting Glu, TG, LDL-C and HDL-C, the association between PG(36:3) and cognitive decline was no longer statistically significant, while the odds ratio (OR) directions and significance of PE(O-40:5), LPC(18:3), PI(38:2) and PA(39:4) did not change.

After adjustment, the risks of cognitive decline increased by 9.05 (OR = 10.05, 95%CI: 1.43, 70.87) times, 6.52 (OR = 7.52, 95%CI: 1.30, 43.48) times, 42.96 (OR = 43.96, 95%CI: 2.61, 741.53) times, 34.29 (OR = 35.29, 95%CI: 4.97, 250.34) times in participants of the Q4 groups of PE(O-40:5), LPC(18:3), PI(38:2) and PA(39:4), respectively. The results of the continuous-analysis showed that for every SD increase in the concentration of PE(O-40:5), LPC(18:3), PI(38:2) and PA(39:4), the risk of cognitive decline increased by 1.43 (OR = 2.43; 95%CI: 1.15, 5.14), 1.20 (OR = 2.20, 95%CI: 1.11, 4.38), 3.13 (OR = 4.13, 95%CI: 1.43, 11.93) and 3.08 (OR = 4.08, 95%CI: 1.88, 8.84) respectively.

**Figure 2:**
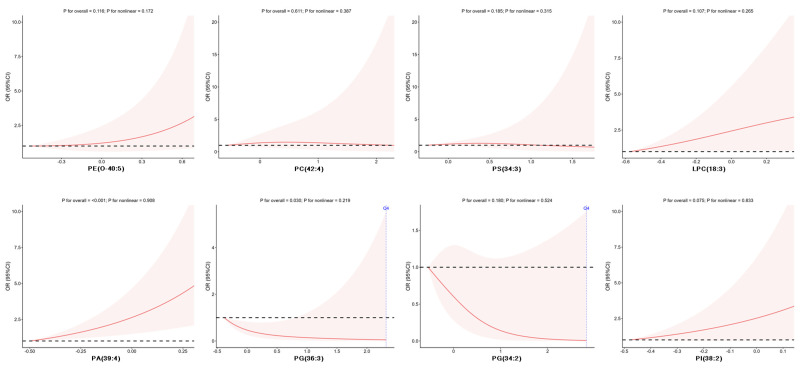
Dose–response associations of phospholipid biomarkers with cognitive decline (adjusted sex, age, educational attainment, smoking status, physical activity, sleep duration, hypertension condition, Glu, TG, LDL-C and HDL-C); black dashed line: reference line for odds ratios equal to 1; solid red lines: odds ratios; pale red shaded area: 95% confidence interval.

The dose–response relationships between eight phospholipid molecules and cognitive decline were shown in Figure 2. Negative linear correlations were observed between the concentrations of PG(36:3) and PG(34:2) and cognitive decline OR value. As the concentrations of these phospholipid molecules increased, the OR values of cognitive decline decreased continuously. And the concentrations of PE(O-40:5), LPC(18:3), PA(39:4), and PI(38:2) showed a positive linear correlation with cognitive decline OR values.

### 3.5. Prediction of Cognitive Decline Through Potential Phospholipid Biomerkers

**Figure 3:**
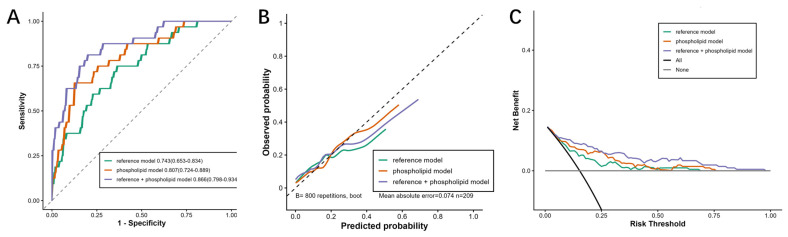
Performance of identified phospholipid molecules in prediction of cognitive decline risk: (**A**) ROC curves, (**B**) calibration curve and (**C**) DCA curves of reference model (including sex, age, educational attainment, smoking status, physical activity, sleep duration, hypertension condition, fasting Glu, TG, LDL-C and HDL-C), phospholipids model (including PE(O-40:5), PC(42:4), PS(34:3), LPC(18:3), PA(39:4), PG(36:3), PG(34:2) and PI(38:2)) and reference + phospholipids model.

The predictive value of the eight phospholipid molecules for cognitive decline was further assessed (Figure 3 and Table S6). Compared with the reference model that only included sex, age, educational attainment, smoking status, physical activity, sleep duration, hypertension condition, fasting Glu, TG, LDL-C and HDL-C, the ROC curves showed that the plasma phospholipid biomarker panel had higher accuracy and better discriminative power for prediction of cognitive decline. By adding the eight plasma phospholipid molecules to the reference model, a significant improvement of AUC was observed from 0.743 (reference model) to 0.866 (reference model + eight plasma phospholipid molecules). DeLong’s tests between the AUCs of these two models displayed that the difference was statistically significant (*p* < 0.05). The calculated NRI and IDI of models indicated the strengthened performance of the model in comparison with basic models (*p* < 0.05). Moreover, the DCA curve demonstrated a higher net benefit of the model for cognitive decline within the threshold probability range of 0 to 1.

## 4. Discussion

To date, very limited longitudinal studies have investigated phospholipid–cognitive decline associations, and these were mainly conducted in European and American populations [13]. To our knowledge, this is the first study that evaluated the association between full-scale phospholipidome and cognitive risk in a cohort study from Chinese population. By applying a targeted phospholipidomic approach to measure 229 phospholipid molecular species in plasma samples, a phospholipid panel including eight molecules was identified relating with cognitive decline through machine learning, and four phospholipid molecules among them were significantly associated with elevated risk of cognitive decline independent of established risk factors in a multivariate regression model, implying that phospholipid perturbations could precede and precipitate the onset of cognitive decline.

Of the eight phospholipids, PG(36:3) was negatively associated with risk of cognitive decline with statistical significance under adjustment of sex, age and lifestyle parameters. But the statistical significances disappeared with adjustment of hypertension, blood glucose and blood lipid, indicating that the relationship between PG(36:3) and cognition might come from hypertension, diabetes or dyslipidemia. In a cross-sectional study based on an Australian population, a similar result was reported that a PG-rich lipid molecular module, including PG (36:1), PG (38:1), and PG (38:2), was associated with higher global cognitive performance, and partially explained the relationship between waist–hip ratio and global cognition, suggesting that PG species may act as intermediaries between central adiposity and cognitive health [14]. Meanwhile, PG(36:2) was reported to exert an anti-inflammatory effect on the innate immune system by inhibiting the activation of Toll-like receptors, and enhance macrophage mitochondrial function, which could counteract the increased oxidative stress and the enhanced inflammation caused by chronic diabetic [15]. Thus, PG might be one of the mediators between obesity-related chronic diseases and cognitive function.

Moreover, the association between plasmalogens and cognitive disorder has been reported in several previous studies. For instance, the concentrations of PE(P-38:6) and PE(P-40:6) were found significantly reduced in the serum of Alzheimer’s disease (AD) patients [16,17], and the AD patients with a deficiency of plasmalogens in the serum developed cognitive decline after one year [7]. In our previous research, the concentration of 13 plasmalogen molecules in plasma was also found to be positively associated with MoCA scores [18]. As important components of cellular membranes, plasmalogens were considered to organize the detergent-resistant microdomains, which are important for cellular signaling and neurotransmitter vesicular fusion [19]. Additionally, plasmalogens are considered to be radical scavengers since the vinyl ether bond has high sensitivity to oxygen free radicals, and deficiency of plasmalogens improves vulnerability to ROS and striatal dopamine loss in mice brain [20,21]. Although the machine learning model selected a plasmalogen molecule in present study, the selected frequency of PE(P-44.5) under bootstrap was lower than 0.5, indicating it has lower stability as a potential biomarker. Instead, an ether phospholipid, PE(O-40:5), was found to be negatively associated with cognitive decline risk with significance. Since the desaturation of ether phospholipids under the catalyzation of plasmanylethanolamine desaturase (PEDS) to form vinyl ether bond is considered to be the final step in biosynthesis of plasmalogens [22], the higher level of ether phospholipid biomarkers in plasma might indicate synthesis disorder of plasmalogens at a very early stage.

In addition, the augmented levels of LPC(18:3) and PA(39:4) were both strongly associated with a higher risk of cognitive decline according to the present study, indicating that the degradation of phospholipids might be a key factor in the development of cognitive impairment. For lyso-phospholipids, similar results has been reported in several horizontal studies, like Li et al., who reported a decrement of LPC(16:0), LPC(18:0), LPC(18:1), LPC(18:2) and LPC(20:4) level in plasma of AD patients compared with control [23], Wood et al. reported a higher concentration of LPE(16:0), LPE(18:1) and LPG(18:1) in plasma of mild AD patients compared with control [24], and our previous research found plasma concentration of LPC(16:1), LPC(22:0) and LPC(24:0) negatively associated with MoCA scores. The capacity of free LPCs to increase cytosolic Ca2+ and activate inflammatory signaling pathways [25] might explain the negative correlation between lyso-phospholipids and cognitive function to a certain extent. However, more targeted research is required to explore whether different lyso-phospholipids identified from different studies have a heterogeneous mechanism. For phosphatidic acids, evidence in mice showed that enhanced PLD activity mediated oligomeric Aβ’s neurotoxic effect in cultured neurons, and bring perturbation of PA [26], which could help explain the relationship between PA(39:4) and cognitive decline risk. But the PA molecules with specific perturbations reported in animal studies still differ from the PA biomarker found in present studies, which also requires more precise pathological studies.

At last, phosphatidylinositol-3-phosphate (PI3P), a kind of phosphorylated PIs, participated in the sorting and processing of amyloid precursor protein through the endosomal system, and is selectively deficient in brain tissue of AD patients [27]. But very limited results have been reported about the relationship of PIs and cognitive function; similarly to our results, Zhang et al. reported that increased levels of PI(38:5) were associated with an elevated risk of dementia with Lewy bodies based on a Mendelian randomization study [28], which is the opposite of the effect of PI3P. Whether the relationship between PI levels and cognition is a feedback indicator of PI phosphorylation inhibition and PI3P downregulation is currently unclear, and further mechanistic research in this area is necessary.

Nevertheless, there are still several limitations of the protocol of the current study, which have to be taken into consideration. First, this cohort study has a limited sample size and observation time, which might bring bias in statistical analysis. Second, the participants included in this study were volunteered from three communities, but not randomly selected based on population, which might bring selection bias. Third, although we adjusted for some covariates during the data analysis, residual confounding was still possible. Fourth, an external cohort validation of the predictive efficacy of these phospholipid biomarkers is not involved, which makes the clinical value unknown.

## 5. Conclusions

In conclusion, detailed phospholipid molecular profiles were identified and quantified in a cohort study with 209 participants followed for one year. A biomarker panel including eight phospholipid molecular species related to cognitive decline was selected through machine learning. And four of these potential biomarkers, including PE(O-40:5), LPC(18:3), PI(38:2) and PA(39:4), were further proved to be significantly associated with the risk of cognitive decline through a logistic regression model with stepwise control of demographic factors, lifestyle, hypertension, fasting blood glucose and blood lipid parameters, indicating that the accumulation of ether phospholipid and PI and the degradation of phospholipids might participate in the process of cognitive decline. By adding the eight phospholipid biomarkers selected in the present study to a reference model that includes adjusted sex, age, educational attainment, smoking status, physical activity, sleep duration, hypertension condition, fasting Glu, TG, LDL-C and HDL-C, the AUC value of the predictive model could be improved from 0.743 to 0.866. This provided a possible auxiliary screening tool for the early detection of cognitive impairment in the elderly.

## Figures and Tables

**Table 1:** The baseline characteristics of the study participants with or without cognitive decline.

Characteristic	Total	Cognitive Decline		*p*-Value
		Cases	Non-Cases	
Participants, *n* [%]	209 [100.0]	32 [15.31]	177 [84.69]	——
Female, *n* [%]	138 [66.0]	24 [75]	114 [64.4]	0.336
Age (years), mean [SD ^1^]	65.8 [4.5]	65.8 [5.2]	65.8 [4.4]	0.955
Educational attainment, *n* [%]				0.063
Non-literate	9 [4.3]	4 [12.5]	5 [2.8]	
Primary school	46 [22.0]	8 [25.0]	38 [21.5]	
Middle school	126 [60.3]	15 [46.9]	111 [62.7]	
High school or higher	28 [13.4]	5 [15.6]	23 [13.0]	
Smoking, *n* [%]				0.258
Non-smoker	178 [85.2]	26 [81.2]	152 [85.9]	
Former smoker	13 [6.2]	4 [12.5]	9 [5.1]	
Current smoker	18 [8.6]	2 [6.2]	16 [9.0]	
Physical activity, *n* [%]				0.757
Do not participate	28 [13.4]	6 [13.6]	22 [13.3]	
2–3 times a month	20 [9.6]	6 [13.6]	14 [8.5]	
3–4 times a week	20 [9.6]	4 [9.1]	16 [9.7]	
≥1 time a day	141 [67.5]	28 [63.6]	113 [68.5]	
Sleep duration (h/d), mean [SD]	6.9 [1.3]	6.7 [1.2]	6.9 [1.3]	0.417
Hypertension, *n* [%]				0.106
No	109 [51.0]	22 [68.8]	87 [49.2]	
Yes	96 [45.9]	10 [31.2]	86 [48.6]	
Unknown	4 [1.9]	0 [0.0]	4 [2.3]	
Diabetes mellitus ^2^, *n* [%]				0.5867
No	187 [89.5]	30 [93.8]	157 [88.7]	
Yes	22 [10.5]	2 [ 6.2]	20 [11.3]	
Dyslipidemia ^2^, *n* [%]				0.4188
No	127 [60.8]	22 [68.8]	105 [59.3]	
Yes	82 [39.2]	10 [31.2]	72 [40.7]	
Glu (mmol/L), mean [SD]	5.78 [1.57]	5.61 [1.32]	5.82 [1.61]	0.485
TG (mmol/L), mean [SD]	1.5 [0.9]	1.4 [0.8]	1.6 [0.9]	0.327
TC (mmol/L), mean [SD]	5.1 [1.0]	5.2 [1.2]	5.1 [0.9]	0.612
LDL-C (mmol/L), mean [SD]	3.2 [0.9]	3.2 [1.0]	3.1 [0.9]	0.573
HDL-C (mmol/L), mean [SD]	1.2 [0.3]	1.19 [0.3]	1.3 [0.3]	0.051
MoCA score at baseline, mean [SD]	23.1 [2.6]	23.3 [2.5]	24.8 [2.7]	0.003
MoCA score after 1 year, mean [SD]	24.6 [3.1]	25.3 [2.6]	20.7 [2.8]	<0.001

^1^ SD: standard deviation. ^2^ No participant was using drugs that regulate lipid metabolism (including statins, aspirin, GLP-1 receptor agonists, thiazolidinediones, metformin and traditional Chinese medicines for regulating blood lipids) at baseline.

**Table 2:** Baseline concentration (mg/L) of phospholipid classes in plasma of participants with or without cognitive decline (mean [SD ^1^]).

Classes	Molecular Species [n]	Total	Cognitive Decline		*p*-Value
			Cases	Non-Cases	
PE	55	44.9 [16.9]	45.3 [18.7]	44.8 [16.5]	0.865
LPE	11	4.7 [1.6]	4.8 [1.8]	4.7 [1.6]	0.900
PC	62	1439.0 [337.3]	1485.8 [393.3]	1425.7 [367.7]	0.367
LPC	22	151.0 [53.2]	164.4 [61.7]	147.2 [50.1]	0.172
PS	22	2.4 [3.4]	2.2 [2.6]	2.5 [3.7]	0.275
PG	5	0.7 [0.4]	0.7 [0.3]	0.7 [0.4]	0.431
PI	19	44.3 [19.6]	46.7 [21.3]	43.6 [19.1]	0.487
PA	3	0.5 [0.3]	0.5 [0.3]	0.4 [0.3]	0.430
SM	30	392.4 [130.8]	423.7 [141.7]	383.5 [126.7]	0.082

^1^ SD: standard deviation.

**Table 3:** Associations (ORs [95% CIs] ^1^) between phospholipid biomarkers with cognitive decline.

Model ^2^	OR for Quartiles				*P*_trand_	OR per SD	*p*-Value
	Q1	Q2	Q3	Q4			
PE(O-40.5)							
Model 1	1	1.68 (1.12, 2.52)	2.52 (1.22, 5.20)	7.44 (1.55, 35.67)	0.1583	2.16 (1.18, 3.95)	0.013
Model 2	1	1.60 (1.03, 2.50)	2.32 (1.05, 5.14)	6.21 (1.11, 34.88)	0.2535	2.02 (1.04, 3.92)	0.039
Model 3	1	1.82 (1.10, 3.01)	2.90 (1.18, 7.13)	10.05 (1.43, 70.87)	0.3636	2.43 (1.15, 5.14)	0.022
PC(42:4)							
Model 1	1	1.15 (0.88, 1.49)	1.39 (0.74, 2.60)	2.23 (0.48, 10.30)	0.4368	1.30 (0.79, 2.12)	0.305
Model 2	1	1.19 (0.90, 1.58)	1.51 (0.77, 2.98)	2.75 (0.52, 14.39)	0.2914	1.39 (0.81, 2.36)	0.233
Model 3	1	1.31 (0.95, 1.80)	1.91 (0.88, 4.11)	4.82 (0.74, 31.45)	0.3840	1.66 (0.91, 3.04)	0.102
LPC(18:3)							
Model 1	1	1.72 (1.18, 2.52)	3.08 (1.40, 6.77)	8.53 (1.89, 38.44)	0.0500	2.31 (1.28, 4.17)	0.006
Model 2	1	1.59 (1.06, 2.40)	2.63 (1.13, 6.12)	6.31 (1.26, 31.66)	0.1863	2.06 (1.09, 3.87)	0.026
Model 3	1	1.67 (1.07, 2.60)	2.88 (1.15, 7.23)	7.52 (1.30, 43.48)	0.2993	2.20 (1.11, 4.38)	0.026
PS(34:3)							
Model 1	1	1.10 (0.85, 1.43)	1.18 (0.75, 1.88)	1.49 (0.50, 4.41)	0.8200	1.21 (0.72, 2.01)	0.473
Model 2	1	1.18 (0.88, 1.57)	1.33 (0.80, 2.22)	1.96 (0.59, 6.55)	0.9681	1.37 (0.78, 2.42)	0.275
Model 3	1	1.14 (0.84, 1.55)	1.25 (0.73, 2.16)	1.71 (0.47, 6.15)	0.7822	1.29 (0.70, 2.35)	0.415
PG(36:3)							
Model 1	1	0.71 (0.53, 0.95)	0.40 (0.18, 0.87)	0.07 (0.01, 0.66)	0.1113	0.38 (0.17, 0.86)	0.021
Model 2	1	0.72 (0.52, 1.00)	0.42 (0.18, 0.99)	0.08 (0.01, 0.98)	0.2293	0.39 (0.16, 0.99)	0.049
Model 3	1	0.75 (0.53, 1.06)	0.46 (0.18, 1.18)	0.11 (0.01, 1.60)	0.2161	0.44 (0.16, 1.19)	0.108
PG(34:2)							
Model 1	1	0.64 (0.41, 1.01)	0.40 (0.16, 1.01)	0.08 (0.01, 1.04)	0.1507	0.47 (0.22, 1.01)	0.055
Model 2	1	0.61 (0.36, 1.03)	0.36 (0.12, 1.07)	0.06 (0.00, 1.21)	0.3913	0.43 (0.18, 1.06)	0.068
Model 3	1	0.59 (0.33, 1.07)	0.34 (0.10, 1.15)	0.05 (0.00, 1.47)	0.2229	0.41 (0.15, 1.12)	0.084
PI(38:2)							
Model 1	1	1.70 (1.10, 2.64)	2.98 (1.21, 7.32)	15.32 (1.62, 144.57)	0.6588	2.78 (1.20, 6.46)	0.018
Model 2	1	1.99 (1.20, 3.29)	4.08 (1.45, 11.46)	33.51 (2.53, 443.56)	0.3836	3.73 (1.42, 9.83)	0.008
Model 3	1	2.09 (1.21, 3.64)	4.55 (1.47, 14.08)	43.96 (2.61, 741.53)	0.4460	4.13 (1.43, 11.93)	0.009
PA(39:4)							
Model 1	1	2.19 (1.38, 3.48)	3.56 (1.68, 7.53)	14.80 (3.02, 72.55)	0.0084	2.90 (1.55, 5.42)	0.001
Model 2	1	2.60 (1.49, 4.56)	4.71 (1.90, 11.68)	26.80 (3.90, 184.14)	0.0026	3.66 (1.71, 7.83)	0.001
Model 3	1	2.82 (1.59, 4.98)	5.36 (2.13, 13.50)	35.29 (4.97, 250.34)	0.0024	4.08 (1.88, 8.84)	0.001

^1^ OR: odds ratio; CI: Confidence interval. ^2^ Model 1: adjusted for sex and age; Model 2: further adjusted for educational attainment, smoking status, physical activity and sleep duration; Model 3: further adjusted for hypertension condition, fasting Glu, TG, LDL-C and HDL-C.

## Data Availability

Data available on request due to restrictions (e.g., privacy, legal or ethical reasons).

## Supplementary Materials

The following are available online at https://www.mdpi.com/article/10.3390/nu18020185/s1, Table S1: Standards and isotope internal standards used for quantification of phospholipid molecular species; Table S2: Quantitative ions of the identified phospholipid molecular species; Table S3: Collinearity diagnostic results of phospholipid molecules included in LASSO regression model; Table S4. Frequencies of the phospholipid molecules selected by LASSO regression under bootstrap; Table S5: Collinearity diagnostic results of covariates; Table S6: Model performance evaluation of different Logistic regression models; Figure S1: Score plots for phospholipid profile in plasma of participants and QC samples based on PCA model; Figure S2: Presentation of the results of the LASSO regression analysis.
