# Supplementary material for: Plasma Phospholipid Biomarkers Related to the Risk of Cognitive Decline in the Elderly: Results from a Cohort Study
Source: Nutrients. 2026 Jan 6;18(2):185. doi: 10.3390/nu18020185 (PMC12844940; doi:10.3390/nu18020185)
Supplement: Supplementary file 1 [file nutrients-18-00185-s001.zip › nutrients-4057685-supplementary.pdf]

Table S1. Standards and isotope internal standards used for quantification of phospholipid molecular species

Standard	Exact Mass	Precursor ion (m/z)	Product ion (m/z)	Internal standard
PC(14:0/14:0)	677.500	678.50	184.10	PC(15:0/18:1) (d7)
PC(15:0/18:1)	745.560	746.55	184.10	
PC(17:0/17:0)	761.593	763.60	184.10	
PC(18:0/18:0)	789.625	790.65	184.10	
PC(18:1/18:1)	785.593	786.60	184.10	
PC(18:2/18:2)	781.562	782.55	184.10	
PC(20:0/20:0)	845.687	846.70	184.10	
PC(22:0/22:0)	901.750	902.75	184.10	
PE(14:0/14:0)	635.453	636.45	495.45	PE(15:0/18:1) (d7)
PE(15:0/18:1)	730.520	704.50	563.50	
PE(17:0/17:0)	719.547	720.55	579.55	
PE(18:0/18:0)	747.578	748.60	607.60	
PE(18:1/18:1)	743.547	744.55	603.55	
PE(18:2/18:2)	739.515	740.50	599.50	
SM(d18:1/12:0)	646.505	647.50	184.10	SM(d18:1/18:1) (d9)
SM(d18:1/16:0)	702.568	703.60	184.10	
SM(d18:1/18:0)	730.599	731.60	184.10	
SM(d18:1/18:1)	728.583	729.60	184.10	
PS(14:0/14:0)	701.424	680.45	495.45	PS(15:0/18:1) (d7)
PS(16:0/16:0)	757.487	736.50	551.50	
PS(15:0/18:1)	769.490	748.50	563.50	
PS(18:0/18:0)	813.550	792.55	607.55	
PS(18:1/18:1)	809.518	788.55	603.55	
PI(16:0/16:0)	827.552	809.50	255.25	PI(15:0/18:1) (d7)
PI(15:0/18:1)	839.550	821.50	241.20	
PI(18:0/18:0)	883.615	865.60	283.30	
PI(18:1/18:1)	879.584	861.55	281.25	
PG(14:0/14:0)	688.429	665.45	227.20	PG(15:0/18:1) (d7)
PG(16:0/16:0)	744.492	721.50	255.25	
PG(15:0/18:1)	756.490	733.50	281.25	
PG(18:0/18:0)	800.554	777.60	283.30	
PG(18:1/18:1)	796.523	773.55	281.25	
PA(14:0/14:0)	614.392	591.40	227.20	PG(15:0/18:1) (d7)
PA(16:0/16:0)	670.455	647.45	255.25	
PA(18:0/18:0)	726.518	703.55	283.30	
PA(18:1/18:1)	722.486	699.50	281.25	
LPC(13:0)	453.286	454.30	184.10	LPC(18:1) (d7)
LPC(17:0)	509.348	510.35	184.10	
LPC(17:1)	507.332	508.35	184.10	
LPC(18:1)	521.394	522.35	184.10	

LPC(19:0)	537.379	538.35	184.10	
LPE(14:0)	425.254	426.25	285.25	LPE(18:1) (d7)
LPE(16:0)	453.286	454.30	313.25	
LPE(18:0)	481.317	482.30	341.30	
LPE(18:1)	479.300	480.30	339.30	

Table S2. Quantitative ions of the identified phospholipid molecular species

No.	Molecular species	Precursor ion (m/z)	Product ion (m/z)	No.	Molecular species	Precursor ion (m/z)	Product ion (m/z)
1	PC(30:0)	706.55	184.1	41	PC(P-34:2)	742.6	184.1
2	PC(32:0)	734.55	184.1	42	PC(P-36:2)	770.6	184.1
3	PC(32:1)	732.55	184.1	43	PC(P-36:3)	768.6	184.1
4	PC(32:2)	730.55	184.1	44	PC(P-36:4)	766.6	184.1
5	PC(34:1)	760.6	184.1	45	PC(P-38:4)	794.6	184.1
6	PC(34:1(OH))	776.6	184.1	46	PC(P-38:5)	792.6	184.1
7	PC(34:2)	758.55	184.1	47	PC(P-38:6)	790.55	184.1
8	PC(34:2(OH))	774.55	184.1	48	PC(P-40:4)	822.65	184.1
9	PC(34:3)	756.55	184.1	49	PC(P-40:5)	820.6	184.1
10	PC(35:1)	774.6	184.1	50	PC(P-40:6)	818.6	184.1
11	PC(35:2)	772.6	184.1	51	PC(P-40:7)	816.6	184.1
12	PC(35:3)	770.55	184.1	52	PC(P-42:3)	852.7	184.1
13	PC(36:1)	788.6	184.1	53	PC(P-42:4)	850.65	184.1
14	PC(36:2)	786.6	184.1	54	PC(P-42:5)	848.65	184.1
15	PC(36:3)	784.6	184.1	55	PC(P-42:6)	846.65	184.1
16	PC(36:4)	782.55	184.1	56	PC(P-44:4)	878.7	184.1
17	PC(36:4(OH))	798.55	184.1	57	PC(P-44:5)	876.7	184.1
18	PC(36:5)	780.55	184.1	58	PC(P-44:6)	874.65	184.1
19	PC(37:2)	800.6	184.1	59	PC(P-44:7)	872.65	184.1
20	PC(37:3)	798.6	184.1	60	PC(P-46:5)	904.7	184.1
21	PC(37:4)	796.6	184.1	61	PC(P-46:6)	902.7	184.1
22	PC(38:2)	814.65	184.1	62	PC(P-46:7)	900.7	184.1
23	PC(38:3)	812.6	184.1	63	PE(34:1)	718.55	577.55
24	PC(38:4)	810.6	184.1	64	PE(34:2)	716.5	575.5
25	PC(38:5)	808.6	184.1	65	PE(34:3)	714.5	573.5
26	PC(38:6)	806.55	184.1	66	PE(36:1)	746.55	605.55
27	PC(40:4)	838.65	184.1	67	PE(36:2)	744.55	603.55
28	PC(40:5)	836.6	184.1	68	PE(36:3)	742.55	601.55
29	PC(40:6)	834.6	184.1	69	PE(36:4)	740.5	599.5
30	PC(40:7)	832.6	184.1	70	PE(38:3)	770.55	629.55
31	PC(40:8)	830.55	184.1	71	PE(38:4)	768.55	627.55
32	PC(42:4)	866.65	184.1	72	PE(38:5)	766.55	625.55
33	PC(42:5)	864.65	184.1	73	PE(38:6)	764.5	623.5
34	PC(42:6)	862.65	184.1	74	PE(38:7)	762.5	621.5
35	PC(42:7)	860.6	184.1	75	PE(40:5)	794.55	653.55
36	PC(42:8)	858.6	184.1	76	PE(40:6)	792.55	651.55
37	PC(42:9)	856.6	184.1	77	PE(40:7)	790.55	649.55
38	PC(44:4)	894.7	184.1	78	PE(40:8)	788.5	647.5
39	PC(O-32:0)	720.6	184.1	79	PE(O-34:1)	704.55	563.55
40	PC(P-34:1)	744.6	184.1	80	PE(O-34:2)	702.55	561.55

No.	Molecular species	Precursor ion (m/z)	Product ion (m/z)	No.	Molecular species	Precursor ion (m/z)	Product ion (m/z)
81	PE(O-36:2)	730.6	589.6	122	SM(d34:1)	703.6	184.1
82	PE(O-36:3)	728.55	587.55	123	SM(d34:2)	701.55	184.1
83	PE(O-36:4)	726.55	585.55	124	SM(d35:1)	717.6	184.1
84	PE(O-38:2)	758.6	617.6	125	SM(d35:2)	715.6	184.1
85	PE(O-38:3)	756.6	615.6	126	SM(d36:1)	731.6	184.1
86	PE(O-38:4)	754.6	613.6	127	SM(d36:2)	729.6	184.1
87	PE(O-38:5)	752.55	611.55	128	SM(d36:3)	727.6	184.1
88	PE(O-40:4)	782.6	641.6	129	SM(d37:1)	745.6	184.1
89	PE(O-40:5)	780.6	639.6	130	SM(d37:2)	743.6	184.1
90	PE(P-34:1)	702.55	561.55	131	SM(d38:0)	761.65	184.1
91	PE(P-34:2)	700.55	559.55	132	SM(d38:1)	759.65	184.1
92	PE(P-36:1)	730.6	589.6	133	SM(d38:2)	757.6	184.1
93	PE(P-36:2)	728.55	587.55	134	SM(d38:3)	755.6	184.1
94	PE(P-36:3)	726.55	585.55	135	SM(d39:1)	773.65	184.1
95	PE(P-36:4)	724.55	583.55	136	SM(d39:2)	771.65	184.1
96	PE(P-36:5)	722.5	581.5	137	SM(d40:0)	789.7	184.1
97	PE(P-38:4)	752.55	611.55	138	SM(d40:1)	787.65	184.1
98	PE(P-38:5)	750.55	609.55	139	SM(d40:2)	785.65	184.1
99	PE(P-38:6)	748.55	607.55	140	SM(d40:3)	783.65	184.1
100	PE(P-40:4)	780.6	639.6	141	SM(d40:4)	781.6	184.1
101	PE(P-40:5)	778.6	637.6	142	SM(d41:1)	801.7	184.1
102	PE(P-40:6)	776.55	635.55	143	SM(d41:2)	799.65	184.1
103	PE(P-40:7)	774.55	633.55	144	SM(d42:1)	815.7	184.1
104	PE(P-40:8)	772.55	631.55	145	SM(d42:2)	813.7	184.1
105	PE(P-42:4)	808.6	667.6	146	SM(d42:3)	811.65	184.1
106	PE(P-42:5)	806.6	665.6	147	SM(d42:4)	809.65	184.1
107	PE(P-42:6)	804.6	663.6	148	PS(32:0)	736.5	551.5
108	PE(P-42:7)	802.6	661.6	149	PS(34:0)	764.55	579.55
109	PE(P-42:8)	800.55	659.55	150	PS(34:1)	762.55	577.5
110	PE(P-44:4)	836.65	695.65	151	PS(34:3)	758.5	573.5
111	PE(P-44:5)	834.65	693.65	152	PS(36:0)	792.6	607.55
112	PE(P-44:6)	832.6	691.6	153	PS(36:1)	790.55	605.55
113	PE(P-44:7)	830.6	689.6	154	PS(36:2)	788.55	603.55
114	PE(P-44:8)	828.6	687.6	155	PS(36:4)	784.5	599.5
115	PE(P-46:6)	860.65	719.65	156	PS(36:5)	782.5	597.5
116	PE(P-46:7)	858.65	717.65	157	PS(38:2)	816.6	631.55
117	PE(P-46:8)	856.6	715.6	158	PS(38:3)	814.55	629.55
118	SM(d32:1)	675.55	184.1	159	PS(38:4)	812.55	627.55
119	SM(d32:2)	673.55	184.1	160	PS(38:5)	810.55	625.5
120	SM(d33:1)	689.55	184.1	161	PS(38:6)	808.5	623.5
121	SM(d34:0)	705.6	184.1	162	PS(39:3)	828.6	643.55

No.	Molecular species	Precursor ion (m/z)	Product ion (m/z)	No.	Molecular species	Precursor ion (m/z)	Product ion (m/z)
163	PS(39:4)	826.55	641.55	194	PA(38:5)	721.5	303.25
							283.25
							255.25
164	PS(39:5)	824.55	639.55	195	PA(39:4)	737.5	303.25
165	PS(40:3)	842.6	657.6	196	PA(40:7)	745.5	327.25
166	PS(40:4)	840.6	655.55	197	LPC(14:0)	468.3	184.1
167	PS(40:5)	838.55	653.55	198	LPC(16:0)	496.35	184.1
168	PS(40:6)	836.55	651.55	199	LPC(16:1)	494.35	184.1
169	PS(40:7)	834.55	649.5	200	LPC(17:0)	510.35	184.1
170	PI(32:1)	807.5	255.25	201	LPC(18:0)	524.35	184.1
171	PI(34:0)	837.55	255.25	202	LPC(18:1)	522.35	184.1
172	PI(34:1)	835.55	255.25	203	LPC(18:2)	520.35	184.1
173	PI(34:2)	833.5	255.25	204	LPC(18:3)	518.35	184.1
174	PI(35:2)	847.55	269.25	205	LPC(19:0)	538.4	184.1
175	PI(36:1)	863.55	283.25	206	LPC(20:0)	552.4	184.1
176	PI(36:2)	861.55	281.25	207	LPC(20:1)	550.4	184.1
			283.25				
177	PI(36:3)	859.55	283.25	208	LPC(20:2)	548.35	184.1
			281.25				
			255.25				
178	PI(36:4)	857.5	255.25	209	LPC(20:3)	546.35	184.1
179	PI(37:4)	871.55	269.25	210	LPC(20:4)	544.35	184.1
180	PI(38:2)	889.6	283.25	211	LPC(20:5)	542.35	184.1
181	PI(38:3)	887.55	283.25	212	LPC(22:0)	580.45	184.1
182	PI(38:4)	885.55	283.25	213	LPC(22:4)	572.35	184.1
183	PI(38:5)	883.55	283.25	214	LPC(22:5)	570.35	184.1
184	PI(38:6)	881.5	255.25	215	LPC(22:6)	568.35	184.1
			281.25				
185	PI(39:4)	899.55	297.3	216	LPC(24:0)	608.45	184.1
			269.25				
186	PI(40:4)	913.6	311.3	217	LPC(P-16:0)	480.35	184.1
			283.25				
187	PI(40:5)	911.55	283.25	218	LPC(P-18:0)	508.4	184.1
188	PI(40:6)	909.55	283.25	219	LPE(16:0)	454.3	313.3
189	PG(34:1)	747.5	281.25	220	LPE(18:0)	482.35	341.35
190	PG(34:2)	745.5	279.25	221	LPE(18:1)	480.3	339.3
191	PG(36:1)	775.55	281.25	222	LPE(18:2)	478.3	337.3
192	PG(36:2)	773.55	307.25	223	LPE(20:2)	506.35	365.35
			279.25				
			281.25				
193	PG(36:3)	771.5	279.25	224	LPE(20:3)	504.3	363.3

No.	Molecular species	Precursor ion (m/z)	Product ion (m/z)
225	LPE(20:4)	502.3	361.3
226	LPE(22:4)	530.35	389.35
227	LPE(22:5)	528.3	387.3
228	LPE(22:6)	526.3	385.3
229	LPE(24:0)	566.4	425.4

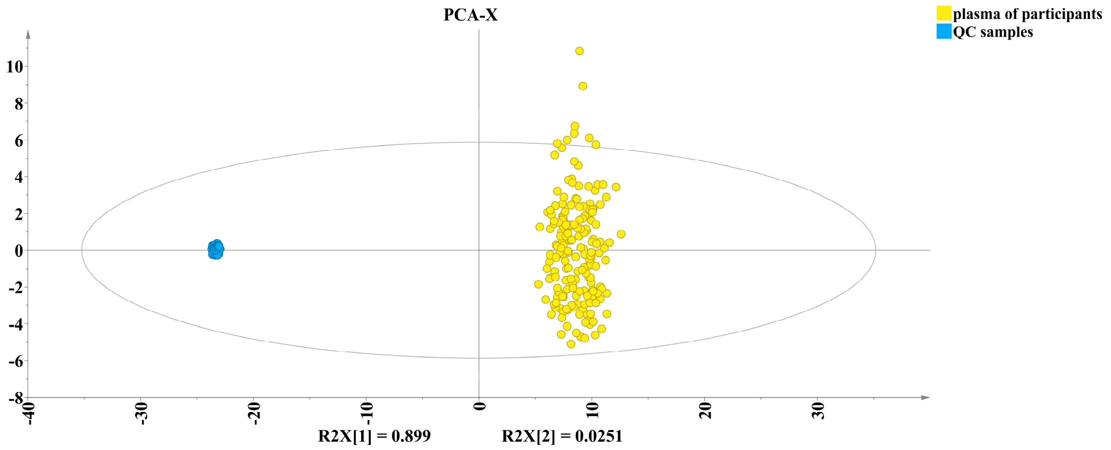

Figure S1. Score plots for phospholipid profile in plasma of participants and QC samples based on PCA model

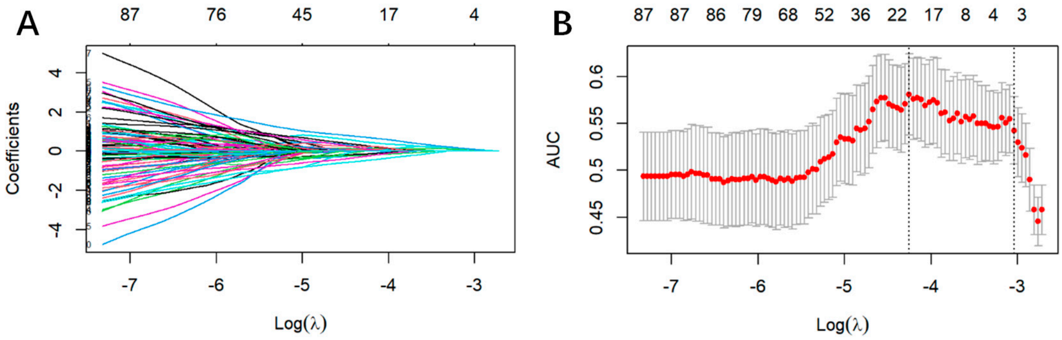

Figure S2. Presentation of the results of the LASSO regression analysis. (A) LASSO regression model screening variable trajectories; (B) LASSO Regression Model Factor Selection: Left dashed line represents the optimal lambda value (lambda·min=0.01421212), while the right dashed line marks the lambda value within one standard error of the optimal (lambda.1se=0.04763339)

Table S3. Collinearity diagnostic results of phospholipid molecules included in LASSO regression model

Variable	Variance Inflation Factor
PE(P-44:5)	3.378
PE(O-40:5)	3.312
PE(36:3)	3.612
PC(42:4)	2.540
PC(35:1)	13.082
PC(O-32:0)	3.641
SM(d36:2)	6.295
SM(d32:2)	5.063
PS(34:3)	1.477
PS(40:6)	1.950
LPC(20:2)	4.629
LPC(18:3)	3.693
PA(39:4)	2.038
PG(36:3)	3.749
PG(34:2)	3.462
PI(38:2)	5.564
PI(36:1)	6.253

Table S4. Frequencies of the phospholipid molecules selected by LASSO regression under bootstrap

Variable	Frequency
PA(39:4)	0.97875
PG(36:3)	0.85375
PE(O-40:5)	0.75000
LPC(18:3)	0.74625
PG(34:2)	0.71250
PS(34:3)	0.64375
PC(42:4)	0.56000
PI(38:2)	0.55125
PE(P-44:5)	0.44750
PS(40:6)	0.43875
SM(d32:2)	0.40875
PC(O-32:0)	0.40125
PE(36:3)	0.39875
LPC(20:2)	0.35875
PI(36:1)	0.25875
SM(d36:2)	0.24875
PC(35:1)	0.13875

Table S5. Collinearity diagnostic results of covariates

Variable	Variance Inflation Factor
sex	1.237
Age	1.044
Educational attainment	1.050
Smoking status	1.094
Sleep duration	1.054
Physical activity	1.039
Hypertension condition	1.043
Glu	1.109
TG	1.980
LDL-C	3.638
HDL-C	1.879

Table S6. Model performance evaluation of different Logistic regression models

Model	AUC	DeLong's test P	Continuous NRI Estimate (95%CI)	*P*	IDI Estimate (95%CI)	*P*	Calibration χ^2	Calibration *P*
Reference	0.743						6.947	0.542
Phospholipids	0.807	0.252	0.210 (0.035, 0.364)	0.014	0.078 (0.004, 0.150)	0.039	12.958	0.113
Reference+Phospholipids	0.866	<0.001	0.490 (0.306, 0.656)	<0.001	0.192 (0.122, 0.261)	<0.001	13.972	0.082

The reference model includes sex, age, educational attainment, smoking status, physical activity, sleep duration, hypertension condition, fasting Glu, TG, LDL-C and HDL-C. Phospholipids model incudes PE(O-40:5), PC(42:4), PS(34:3), LPC(18:3), PA(39:4), PG(36:3), PG(34:2) and PI(38:2). NRI, net reclassification improvement; IDI, integrated discrimination improvement.
